# Integrated analysis of gene expression by association rules discovery

**DOI:** 10.1186/1471-2105-7-54

**Published:** 2006-02-07

**Authors:** Pedro Carmona-Saez, Monica Chagoyen, Andres Rodriguez, Oswaldo Trelles, Jose M Carazo, Alberto Pascual-Montano

**Affiliations:** 1 BioComputing Unit, National Center for Biotechnology (CNB-CSIC), Cantoblanco, 28049, Madrid, Spain; 2 Computer Architecture Department, Universidad de Málaga, 29080, Málaga, Spain; 3 Computer Architecture and System Engineering Department, Facultad de CC Físicas, Universidad Complutense de Madrid, 28040, Madrid, Spain

## Abstract

**Background:**

Microarray technology is generating huge amounts of data about the expression level of thousands of genes, or even whole genomes, across different experimental conditions. To extract biological knowledge, and to fully understand such datasets, it is essential to include external biological information about genes and gene products to the analysis of expression data. However, most of the current approaches to analyze microarray datasets are mainly focused on the analysis of experimental data, and external biological information is incorporated as a posterior process.

**Results:**

In this study we present a method for the integrative analysis of microarray data based on the Association Rules Discovery data mining technique. The approach integrates gene annotations and expression data to discover intrinsic associations among both data sources based on co-occurrence patterns. We applied the proposed methodology to the analysis of gene expression datasets in which genes were annotated with metabolic pathways, transcriptional regulators and Gene Ontology categories. Automatically extracted associations revealed significant relationships among these gene attributes and expression patterns, where many of them are clearly supported by recently reported work.

**Conclusion:**

The integration of external biological information and gene expression data can provide insights about the biological processes associated to gene expression programs. In this paper we show that the proposed methodology is able to integrate multiple gene annotations and expression data in the same analytic framework and extract meaningful associations among heterogeneous sources of data. An implementation of the method is included in the Eng*e*ne software package.

## Background

DNA microarray technology is a powerful method for exploring biological processes on a genomic scale. This high-throughput technique allows researchers to simultaneously monitor the expression level of thousands of genes, or even whole genomes, in a single experiment. It has been successfully used in many contexts, such as tumor classification, drug discovery or temporal analysis of cell behavior [1]. One of the great potentials of this method is that the datasets generated contain global information about the biological processes that govern cell behavior. Nevertheless, in order to interpret gene expression patterns and to discern the underlying biological mechanisms, it is essential to include external information about genes and gene products in the analysis of such datasets.

A key task to derive biological knowledge from gene expression data is to detect the presence of sets of genes that share a similar expression pattern and common biological properties, such as function or regulatory mechanism. Current approaches to analyze microarray data in this line are frequently based on the application of clustering algorithms in order to establish sets of co-expressed genes. Nevertheless, these algorithms do not incorporate available information about genes and gene products and they just take into account experimental measurements. Therefore, each set of co-clustered genes has to be further examined with the aim of discovering common biological connections among them. In this way, biological information is incorporated as a subsequent process to the analysis of expression data.

Although widely used, this type of approach shows some well-known drawbacks:

(i) The underlying assumption in this analysis is that genes sharing similar expression profiles also share similar biological properties. Nevertheless, simultaneously expressed genes may not always share the same function or regulatory mechanism. Even when similar expression patterns are related to similar biological roles, discovering these biological connections among co-expressed genes is not a trivial task and requires a lot of additional work [2].

(ii) Standard clustering algorithms group genes whose expression levels are similar across all conditions. However, a group of genes involved in the same biological process might only be co-expressed in a small subset of experimental conditions. In this sense, methods that can pull out subsets of genes associated with small subsets of experiments are likely to be useful [3]. Although several approaches have dealt with this problem [4-6], they are mainly focused on finding sets of related genes based only on expression data. Biological knowledge is still incorporated as a subsequent step to expression data analysis.

(iii) Many genes can be conditionally co-expressed with different sets of genes, which may reflect the different biological roles that a gene product can play in the cell [7]. Most of the commonly used clustering algorithms group genes into single clusters, which mask these complex relationships among different sets of conditionally regulated genes.

Consequently, the development of methods able to include external biological information to appropriately analyze and interpret microarray data remains as one of the main challenges in bioinformatics research. Indeed, in the last few years several methods have been introduced to integrate heterogeneous data sources in the context of gene expression data analysis (for a review see [8]).

Association rules discovery technique (ARD) is a data mining method that has been extensively used in many applications to discover associations among subsets of items from large transaction databases. This method detects sets of elements that frequently co-occur in a database and establish relationships between them of the form of *X *→ *Y*, which means that when *X *occurs it is likely that *Y *also occurs. The left hand side of the rule is named antecedent and the right hand side is named consequent. This technique has been recently proposed to the analysis of gene expression data [9-13] in order to extract associations and relationships among subsets of genes of the form: {[+]*gene A *→[+]*gene B*, [+]*geneC*}, meaning that when *gene A *is over-expressed it is also very likely to observe an over-expression of *gene B *and *gene C*. This approach avoids some of the existing drawbacks of standard clustering algorithms and has been proved to be successful in extracting new and informative gene relationships. Nevertheless, these previous works focus only on the analysis of gene expression data without incorporating functional annotations or other type of biological knowledge.

In this work we propose an innovative application of ARD for the integrative analysis of gene expression data. We show that this methodology is able to integrate different types of data in the same analytic framework to uncover significant associations among gene expression profiles and multiple gene annotations based on co-occurrence patterns. The method can be applied to mine annotated gene expression datasets in order to extract associations like the following one: {*cell cycle *→[+]*condition *1, [+]*condition *2, [+]*condition *3, [-]*condition *6}, which means that, in the dataset, a significant number of the genes annotated as "cell cycle" are over-expressed in condition 1, 2 and 3 and under-expressed in condition 6. The significance of these associations is assessed by different quality measures, such as the support (the proportion of annotated genes in the data that are covered by the rule) the confidence (the percentage of genes annotated as "cell cycle" that show the expression pattern defined in the consequent of the rule) and the improvement of the rule (a correlation measure between antecedent and consequent). This approach integrates expression data and biological information to uncover relationships without any pre-established assumption, *i.e*., an association is only reported if there is a significant set of genes that share a biological attribute and a similar expression pattern. The associations are, therefore, intrinsic to the data. In addition, it also offers the advantage that each gene can be annotated with several topics and all of them will be independently taken into account to discover latent relationships. For example, the method is able to extract information about sets of genes that share functionality, transcriptional mechanism and similar expression patterns. Therefore, this type of associations can reveal meaningful connections among biological information of genes and expression patterns (over- or under-expression patterns) that can be very useful for the analysis and interpretation of microarray data.

One of the major limitations of ARD is the large amount of rules that are generated, which becomes a major problem in many applications. This fact has been already pointed out in several studies, where some post-processing pruning methods have been proposed to reduce the number of generated rules. For example, in the context of gene expression, Creighton and Hanash imposed constraints on the size of the rules, extracting only those formed by seven or more genes [10] while Tuzhilin and Adomavicius proposed several post-processing operators for selecting and exploring interesting rules from the whole set [12]. Other related works are more focused on the application of efficient mining methods to detect only significant rules, like for example high confident associations [14]. In this work we have used filter options especially designed to eliminate those associations that are not relevant for the analysis. One of them is based on the observation that many of the rules generated by ARD are intrinsically redundant and therefore can be properly filtered out without losing any relevant information. This option drastically reduces the number of associations to be examined. In addition, we used a statistical test of significance to point out the relevance of the rules generated and to filter out those whose association is not significant.

To illustrate the usefulness of the proposed methodology we show the analysis of two well studied microarray datasets, one is related to the metabolic shift from fermentation to respiration in yeast [15] and the other reports the changes in gene expression of human fibroblasts after serum exposure [16]. We incorporated external information such as metabolic pathways, transcriptional regulators that bind to promoter regions and Gene Ontology (GO) terms to the analysis of expression data.

Using these annotated datasets, the method was able to extract several associations that reveal meaningful information about the biological processes related to these metabolic changes. Many of the associations found by our approach have been recently reported in independent works but others are not well characterized and might be interesting to be further investigated. Our results show that this method can be a very useful tool to integrate the analysis of gene expression data and external biological information in a single process. An implementation of this method is included in the Eng*e*ne™ (Gene-Expression Data Processing and Exploratory Data Analysis) software package [17], freely accessible upon request [18].

## Results

We applied our approach to the analysis of several gene expression datasets integrating different sources of biological information such as metabolic pathways, Gene Ontology annotations or transcriptional regulators. Full results are available as supplementary material in our web site [19]. In particular, in this work we describe in detail the results from the analysis of two well studied microarray datasets. One is related to the metabolic shift from fermentation to respiration in *Saccharomyces cerevisiae *[15] and the other is related to the gene expression program of human fibroblasts after serum exposure [16]. We first describe the detailed analysis of the diauxic shift dataset; illustrate the process of extracting association rules and their interpretation in a biological context. In a second section we describe the main patterns discovered in the serum stimulation dataset.

### Diauxic shift dataset

Briefly, the experiment investigates the temporal program of gene expression accompanying the metabolic shift from fermentation to respiration that occurs when fermenting yeast cells, inoculated into a glucose-rich medium, turn to aerobic utilization of the ethanol produced during the fermentation after the fermentable sugar is exhausted. This dataset contains whole-genome expression levels during this metabolic change. Experiments are numbered from time points one to seven (T1-T7) and correspond to samples harvested at successive two-hour intervals after an initial nine hours of growth.

In this dataset we incorporated external information about metabolic pathways and transcriptional regulators that bind to promoter regions. This annotated dataset was first transformed into a transaction dataset (see Methods) and association rules were then extracted using the constraints that gene annotations appear in the antecedent and gene expression patterns in the consequent. We evaluated our method in two different ways: mining the data using only one of these biological properties and mining the data using both properties together (full results are available in the additional file 1). In the next sections we describe the results of mining the annotated gene expression dataset and the biological interpretation of the associations extracted.

#### Association rules among metabolic pathways and expression patterns

**Figure 1:**
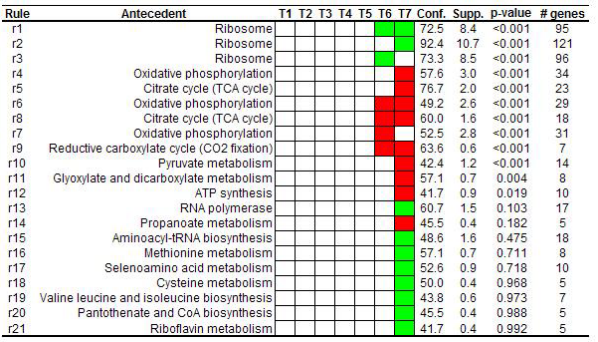
**Rules related to metabolic pathways. **Rules extracted from the diauxic shift dataset using KEGG pathways. To facilitate the visualization, the consequent elements are graphically represented by colored squares. Red color represents over-expression, green color represents under-expression and empty squares represent neither over-expression nor under-expression. For example, the first rule should be {*Ribosome *→[-]T 6, [-]T 7} in the classical representation. Only values for support (supp.), confidence (conf.) and permutation corrected *p*-values are shown for each rule, the rest of measures are reported in the additional files. The last column contains the number of genes covered by each association.

Discovering that most of the genes involved in a specific metabolic pathway are over- or under-expressed in the same experimental conditions provides clues about the biological processes that can be acting under these experimental circumstances. A set of 1126 yeast genes of more than 6000 included in the analysis were associated with at least one pathway from KEGG database [20]. Association rules were extracted with absolute minimum support value of 5 (which correspond to 0.44% of the whole dataset), minimum confidence of 40% and minimum improvement of one, obtaining a total of 40 association rules. As one gene can be involved in more than one pathway, rules containing information about co-occurrences of different pathways in the antecedent were also extracted. Nevertheless, since it is usual to analyze information about individual pathways or biological processes, the *single antecedent *and *redundant *filters were applied (see Methods). 21 association rules passed these filters (Figure 1).

#### Association rules among transcriptional regulators and expression patterns

**Figure 2:**
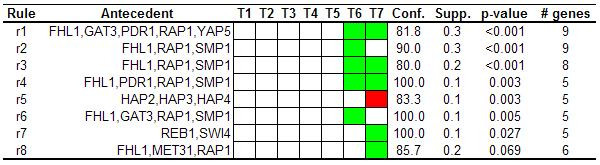
**Rules related to transcriptional regulators. **Rules extracted from the diauxic shift dataset using transcriptional regulators that bind to promoter regions.

Another common approach used to derive biological knowledge from gene expression data is to extract information about transcriptional mechanisms. Promoter regions of co-expressed genes can be analyzed in order to find common upstream sequence motifs [3]. In the last few years, genome-wide location analysis experiments have opened new ways for studying regulatory relationships. These methods are generating an inestimable source of information about physical interactions among transcriptional regulators and DNA regions. Lee *et al*. monitored binding sites for most of the transcriptional regulators encoded in the eukaryote *Saccharomyces cerevisiae *[21]. Using these data, we annotated yeast genes with transcriptional regulators that bound to their promoter region. A total of 3490 genes were annotated with at least one transcriptional regulator in the diauxic shift dataset. Association rules containing transcriptional regulators were extracted using absolute minimum support value of 5 (0.14% of the whole dataset), minimum confidence of 80% and minimum improvement of one, obtaining a final set of 28 associations rules. Different transcriptional regulators can cooperate to regulate gene expression, thus information about putative combinations of transcriptional regulators can provide meaningful insights about transcriptional mechanisms. Consequently, only *redundant *filter was applied, after which only 8 association rules were obtained (Figure 2).

#### Association rules among transcriptional regulators, metabolic pathways and expression patterns

**Figure 3:**
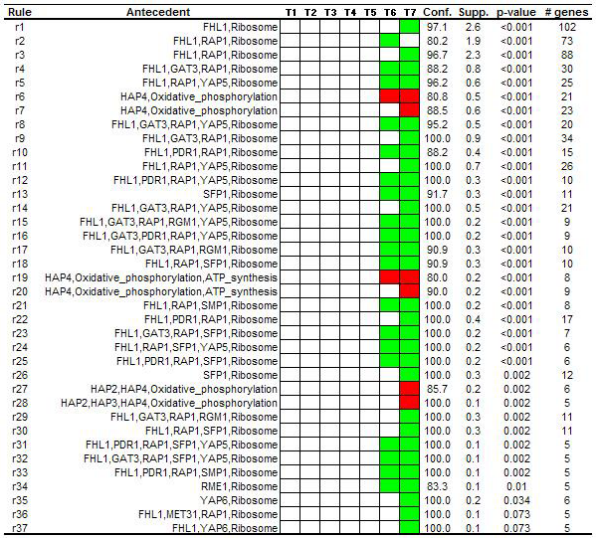
**Rules related to transcriptional regulators and metabolic pathways. **Rules extracted from the diauxic shift dataset using transcriptional regulators and KEGG pathways simultaneously.

Finally, to complete the analysis and to explore the full potential of the method to integrate multiple types of data, we performed a third experiment combining transcriptional regulators and metabolic information. 3882 genes of the dataset were properly annotated and used for the analysis. In this case, association rules relate a transcriptional regulator with a metabolic pathway if there is a significant set of genes that share both characteristics and also show a similar expression pattern. 286 association rules were obtained using absolute minimum support value of 5 (0.13% of the whole dataset), minimum confidence of 80% and minimum improvement of one. In this experiment, only *redundant *filter was applied due to the fact that these types of associations contain more than one item in the antecedent (co-occurrences among pathways and transcriptional regulators). 37 rules whose antecedent contained transcriptional regulators together with metabolic pathways survived the filtering process (Figure 3).

#### Biological significance of the discovered associations

To evaluate the biological significance of the associations that were provided by the method, we should pay attention to the support and confidence values assigned to each rule. The support of a rule indicates the percentage of transactions (annotated genes) that show co-occurrences of a given annotation(s) and a similar expression pattern. In the same way, the confidence value represents the percentage of genes of a given category (represented by the antecedent) that show the expression pattern appearing in the consequent of the rule. In the type of rules proposed in this work, perhaps confidence is the most significant value from the biological point of view. If only a small set of genes are annotated into a very specific category, the support value of the rules containing this annotation will be quite low. Nevertheless, if these rules have a high confidence value, they reveal that this specific biological property is highly associated with the expression pattern that appears in the consequent.

As can be noted, rules extracted from the yeast dataset only revealed marked alterations at time points 6 and 7 of the time course experiment, which is in agreement with the curve of glucose concentration reported in the original paper [15]. This fact indicates that marked changes in gene expression patterns occur at the last time points of the experiment, when glucose was exhausted from the medium. During exponential growth in glucose-rich medium, the global expression profile was remarkably stable. Looking at Figure 1 we can see that there is one rule (rule 3) that shows that more than 70% of all genes annotated as "ribosome" were under-expressed at time point 6 while another rule (rule 2) shows that more than 90% of the genes annotated with this category were under-expressed at time point 7. This increase in confidence (and also in support) value from time point 6 to 7 indicates that an increasing number of ribosomal genes were significantly under-expressed. The association of this pathway, and the under-expression pattern of some genes involved in pathways related to protein and nucleic acid biosynthesis, is in agreement with the observation that yeast cells enter into a non proliferating stationary phase in response to glucose depletion [22].

Rules containing information about metabolic pathways associated to over-expression patterns also revealed meaningful associations. One association (rule 8) shows that 60% of genes involved in "TCA cycle" were mainly over-expressed at time points 6 and 7. This percentage increases at time point 7, when more than 76% of all genes annotated as "TCA cycle" were over-expressed (rule 5). Additionally, other extracted rules also show that genes involved in "glyoxylate and dicarboxylate metabolism" as well as other metabolic pathways related to carbon and energy metabolism were also mainly over-expressed at time point 7 which reflects the main metabolic changes associated to diauxic shift in yeast, manually identified in the original work [15].

In the same way, rules related to transcriptional regulators also provide meaningful associations. DeRisi *et al*., analyzing promoter regions of a set of under-expressed genes in response to glucose depletion, found Rap1-binding motifs in upstream sequences of seven genes that codified for ribosomal proteins [15]. This observation is in concordance with some automatically extracted associations in our analysis that shows that genes whose promoters were bound by the product of *RAP1*, and also by other transcriptional regulators codified by several genes (*FHL1*, *GAT3*, *SMP1*, *PDR1*, *YAP5*), were mainly under-expressed at the two last time points of the time course experiment (Figure 2). Interestingly, in an independent study this set of transcriptional regulators have been related to co-expression of genes involved in ribosome biogenesis [23]. Indeed, when association rules were extracted in order to discover co-occurrences among metabolic pathways and transcriptional regulators, all these transcriptional regulators were associated to "ribosome" (Figure 3). This reveals that promoter regions of genes that codify for ribosomal proteins were bound by this set of transcriptional regulators and, in addition, they were highly repressed in response to glucose depletion.

In the original work, the authors also reported that a set of cytochrome c-related genes, which were over-expressed during diauxic shift, presented *HAP2, HAP3, HAP4 *binding sites in their upstream sequences [15]. As can be seen in Figure 2, one extracted rule reveals that more than 80% of genes that were bound by the products of *HAP2*, *HAP3 *and *HAP4 *were over-expressed at time point 7 of the experiment (rule 5). Looking at Figure 3 we can note that the under-expressed genes whose promoters regions were bound by these transcriptional regulators were mainly involved in "oxidative phosphorylation", the biological process in which *cytochrome c*-related genes are involved.

Although we used a well studied yeast dataset to show the usefulness of our approach for the integrated analysis of gene expression data, we also found interesting associations that have been experimentally confirmed in independent works. Three rules (rule 1, 2 and 3 in Figure 3) show that ribosomal genes whose promoter regions were bound by *RAP1 *and *FHL1 *gene products presented an inhibition pattern in response to nutrient starvation. These three associations were extracted with relative high support values and suggest a connection among *FHL1 *and *RAP1 *and the decrease in ribosomal gene transcription in response to glucose depletion. It is well-known the connection among *RAP1 *and ribosomal gene transcription [24].

Nevertheless, until very recently, little experimental information was available about *FHL1 *beyond the fact that it was implicated in RNA polymerase III function and its mutation causes a lower rRNA content [25]. However, in the last few years important studies have confirmed the connection between this transcriptional regulator and protein ribosome biogenesis [26-29], which is in concordance with the rules obtained by our method. Moreover, in some of these works the authors also comment that binding of Fhl1 to ribosomal gene promoters can be influenced by Rap1 [26,27]. DeRisi *et al*. reported a decrease of *RAP1 *mRNA levels in the cell at about the time of glucose exhaustion [15]. Interestingly, in the analyzed dataset we observed that abundance of *FHL1 *mRNA diminished by two-fold at time point 7 of the experiment, which is the same change showed by *RAP1 *at this time point.

Another association (rule 26 in Figure 3) showed that 100% of genes whose promoter regions were bound by the *SFP1 *gene product and were annotated as "ribosome" were inhibited in response to nutrient starvation. In a recently published work, Marion *et al*. [30] have demonstrated that this transcription factor is released from ribosomal protein gene promoters and ribosomal protein gene transcription is down-regulated in response to changes in nutrient availability. This, and the previously commented associations, clearly support the results obtained by our method and show its potential to find meaningful associations integrating biological information and gene expression data.

### Serum stimulation dataset

Iyer *et al*. monitored the gene expression program of human fibroblast after serum exposure [16]. They added fresh medium containing serum to quiescent fibroblasts and measured the temporal changes in mRNA levels of more than 8000 human genes at 12 times, ranging from 15 min to 24 hours after serum stimulation. From this dataset they established more than 500 genes whose expression changed substantially in response to serum. We used this dataset to extract association rules among Gene Ontology terms and gene expression patterns. The Gene Ontology Consortium has developed a standardized and dynamic vocabulary about gene products in several organisms at three different categories; Molecular Function, Biological Process and Cellular Component [31]. This ontology is one of the most used information sources to categorize and annotate gene products.

We annotated all genes in the array with terms from the three categories of GO. We first used this annotated dataset to extract rules among biological process annotations and expression patterns and in a second analysis we extracted rules among combinations of GO terms from the three categories and expression patterns (full results are available in the additional file 2).

#### Association rules among Biological Process annotations and expression patterns

**Figure 4:**
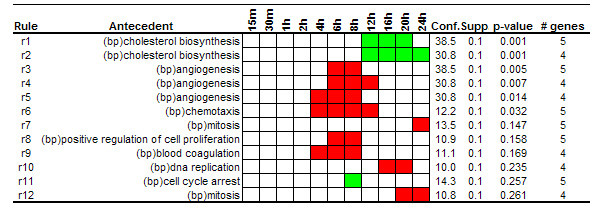
**Rules related to GO Biological Process category. **Rules extracted from the serum stimulation dataset using the Biological Process category of Gene Ontology. Bp in brackets denotes biological process categories.

To get insights into the main biological processes underlying the serum stimulation response along the time course experiment we first extracted association rules among biological process annotations and gene expression patterns. A set of 4092 genes of more than 8000 present in the array were annotated with at least one term from the biological process category. 116 associations were extracted using absolute minimum support value of 4, minimum confidence of 10% and minimum improvement of one. From this set of rules, 12 associations were obtained after applying the *single antecedent *and *redundant *filters (Figure 4). The extracted associations revealed the main biological processes that play relevant roles during the serum response. The biological process of "angiogenesis" was highly associated with over-expression patterns at times ranging from 4 to 12 hours after serum stimulation. This function is also associated to the physiology of wound healing, process that has been associated to the fibroblast serum-response gene expression program [16,32].

A closer look to the extracted rules also revealed that a set of genes annotated as "cholesterol biosynthesis" were associated to under-expression patterns at 12, 16, 20 and 24 hours after serum exposure. In the original work, the authors also reported this observation. They attributed this under-expression to the fact that serum provides cholesterol to fibroblasts. In absence of cholesterol in the medium the cell activates endogenous cholesterol biosynthetic pathways but when external cholesterol is provided genes involved in this pathway are repressed.

#### Association rules among terms from all GO categories and expression patterns

In a second experiment, we have applied our method to find associations among GO terms combinations (from the three categories) and gene expression patterns. 4630 genes were annotated with at least one GO term from the three categories. 192 associations were extracted using absolute minimum support value of 4, minimum confidence of 10% and minimum improvement of one. There are gene ontology terms that although they are related to concrete aspects of the cell physiology they can cover sets of genes involved in disparate biological processes. For example, genes annotated as "cell communication" can be involved in a broad range of cellular processes, or the category "cell proliferation" can cover genes with antagonist effects such as "positive regulation of cell proliferation" or "negative regulation of cell proliferation". In a given dataset, these categories may not be associated to expression patterns because a large number of genes showing diverse expression profiles can belong to these categories. In this case, the combination of annotations can provide additional information for the interpretation of gene expression data.

**Figure 5:**
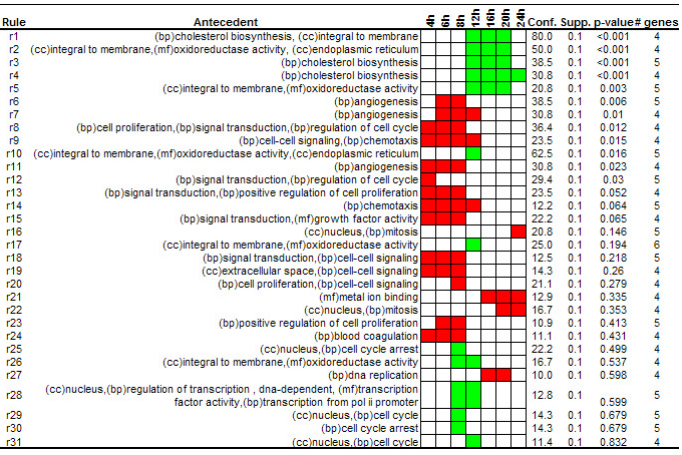
**Rules related to annotations from the three categories of GO. **Rules extracted from the serum stimulation dataset using terms from the three categories of GO. Bp: biological process, cc: cellular component and mf: molecular function. Times corresponding to 15 min., 30 min., 1 hr. and 2 hr. are omitted because there were not significant associations at these time points for the thresholds used to extract association rules.

To test this hypothesis we applied only *redundant *filter and 31 of the initial 192 associations remained for further analysis (Figure 5). As can be noted in Figure 5, many associations are similar to the case in which we used only the biological process category, but there are also interesting associations related to co-occurrences of GO annotations. For example, in the analyzed dataset we did not find a significant association containing the "signal transduction" category. This annotation covered a large number of genes, many of them related to different aspect of the serum response. Nevertheless, we found that genes that were annotated as "signal transduction" and also were annotated with other categories such as "positive regulation of cell proliferation" (rule 13) showed over-expression patterns at times 4, 6 and 8 hours after serum stimulation.

Another association indicates that more than 80% of genes involved in "cholesterol biosynthesis" and annotated as "integral to membrane" (rule 1 in figure 5) are significantly under-expressed at times ranging from 12 to 20 hours. This rule reveals the association previously commented among cholesterol biosynthesis and under-expression patterns after serum exposure but it also provides additional information about some aspects of this process, which is the cellular localization of some proteins involved in this pathway. Many proteins involved in cholesterol biosynthesis and transport are localized in the endoplasmic reticulum, peroxisomes or Golgi apparatus [33,34].

## Discussion

In this paper we present a method for the integrative analysis of microarray data. The approach is based on the association rules discovery technique and is able to automatically extract intrinsic associations among gene annotations and expression patterns. These relationships can provide valuable information for the analysis and interpretation of gene expression datasets. Indeed, one of the main goals in the analysis of microarray data is to uncover connections and relationships among functional annotations and expression patterns. Opposite to other approaches in which biological information is independently incorporated to expression data analysis, our approach integrates both data sources in a single framework to extract associations based on co-occurrence patterns.

The analysis of two gene expression datasets shows the potential of our method to integrate heterogeneous sources of information and gene expression data. In the present analysis, our approach was able to uncover biologically meaningful associations clearly supported by previous studies. Although in this study we integrated gene expression data with three gene properties (metabolic pathways, transcriptional regulators and GO terms), the method can be easily extended to test any type of gene annotation worthy to be evaluated in the context of gene expression, such as sequence motifs or biological keywords. Once genes have been annotated with the biological properties to be analyzed, extracting associations among these annotations and expression patterns becomes an easy and automatic task. In addition, the implementation of the method also allows users to include experimental features, for example the type of tissue, to extract association rules among these features and gene expression.

As we have shown in the results, a relevant characteristic of our approach lies in its ability to integrate different data sources to uncover local relationships based on co-occurrences of sets of annotations and expression patterns. This feature has a clear biological relevance due to the fact that gene expression programs can be associated to different biological factors. Therefore, the integration of different types of biological information is an essential consideration to fully understand the underlying biological processes.

We would like to mention that we found related results when we compared our analyses with others methods that have been applied to the same datasets (see for example [35,36]). Nevertheless, the main advantage of the method we propose is the ability to integrate several gene annotations and gene expression patterns, including over- and under-expression, across several experimental conditions in a single analysis. This feature is important in the analysis of microarray data since in many cases one have to perform two different analyses to discover biological annotations associated to over- and under-expressed patterns. A possible alternative could be the analysis of the enrichment of biological annotations in the whole set of genes, over- and under-expressed [32], but although this approach is able to find terms that are statistically over-represented in a input list, it does not provide any information about the association of multiple categories with over- or under-expressed genes.

As many others methods, ARD requires the selection of an expression threshold to consider a gene (or sets of genes) over- or under-expressed. The selection of expression thresholds is a widely studied problem in microarray data analysis and several tools are available to approach this task that can be used before extracting association rules. Although this issue is out of the scope of this work we want to mention that the use of very restrictive expression thresholds can generate a small number of associations related to high changes in expression levels. Associations related to genes that show less marked changes can be extracted using more relaxed thresholds. In a recent work Pan *et al*. [37] have suggested that "the robustness of biological conclusions made by using microarray analysis should be routinely assessed by examining the validity of the conclusions by using a range of threshold parameters".

One known drawback of ARD is that the number of generated rules is usually very high, even if large values for minimum support and confidence are used. This enormous amount of information is difficult to manually process and usually requires examination of the generated rules to extract those that are more interesting for a particular application. In this work we have tackled this issue by proposing some filtering procedures for eliminating redundant associations which efficiently reduced the number of obtained rules. These filtering options are not only necessary for the human interpretability of the results, but also for obtaining a better quality of the results, since the filtered rules are not informative.

## Conclusion

In this work we have described a methodology for the integrated analysis of microarray data that is able to extract associations among functional annotations and gene expression patterns. The approach is based on the association rules discovery technique and it is included in the Eng*e*ne software package [18], freely available upon request. We hope that the proposed methodology would help the microarray community in the analysis and interpretation of gene expression data.

## Methods

### Definition of association rules

ARD is a data mining technique oriented towards finding associations or correlation relationships among items in transaction datasets. This method extracts sets of items that frequently occur together in the same transaction, and then formulate rules that characterize these relationships.

Making a formal statement of the problem, let *I *= {*i*_1_,*i*_2_,..., *i_n_*} be a set of literals called items. Let *S *be a set of transactions, where each transaction *T *is a set of items such that *T *⊆ *I*. We can now say that a transaction *T *contains a set *X *of items in *I *if *X *⊆ *T*. An association rule is an implication of the form *X *→ *Y*, where *X *⊆ *I*, *Y *⊆ *I *and *X *⋂ *Y *= *φ*. The left hand side of the rule is called antecedent and the right hand side is called consequent. Such rules are usually interpreted as follow: when *X *occurs, it is often the case that *Y *also occurs in the same transaction. This technique has been widely used in Market Basket Analysis to extract associations among products that are frequently sold together in the same transaction (market basket).

Given the association rule *X *→ *Y*, there are two measures that define the quality of the rule:

(i) Its support, which is defined as *P*(*X *⋃ *Y*), that is, the probability that *X *and *Y *appear together.

(ii) Its confidence, which is the conditional probability of *Y *given *X*, and it is

defined as: P(X∪Y)P(X)
 MathType@MTEF@5@5@+=feaafiart1ev1aaatCvAUfKttLearuWrP9MDH5MBPbIqV92AaeXatLxBI9gBaebbnrfifHhDYfgasaacH8akY=wiFfYdH8Gipec8Eeeu0xXdbba9frFj0=OqFfea0dXdd9vqai=hGuQ8kuc9pgc9s8qqaq=dirpe0xb9q8qiLsFr0=vr0=vr0dc8meaabaqaciaacaGaaeqabaqabeGadaaakeaadaWcaaqaaiabdcfaqjabcIcaOiabdIfayjabgQIiilabdMfazjabcMcaPaqaaiabdcfaqjabcIcaOiabdIfayjabcMcaPaaaaaa@37BF@.

Support and onfidence are the most common measures related to a rule and, in many cases, the only ones used to point out the relevance of this one. However, it is important to note that sometimes both of these measures are high, indicating a rule which could be good, and yet still produce a association that is not useful. In other words, associations among uncorrelated elements can be generated using this "support-confidence" framework [38]. This is the case in which the elements of the consequent are very frequent in the transaction database [39]. For example, consider the following rule:

{*A *→ *B*, *C*} support = 60% and confidence = 80%

This rule indicates that 60% of all transactions contain A, B and C, and that 80% of transactions containing A also contain B and C. The above rule looks like a good rule, but it is really not a useful association if B and C are present in 100% of the transactions. Thus, a correlation measure between antecedent and consequent is needed to assess the quality of the rule. In our approach we have implemented the improvement (also known as lift) value for association rules, which is defined as: P(X∪Y)P(X)∗P(Y)
 MathType@MTEF@5@5@+=feaafiart1ev1aaatCvAUfKttLearuWrP9MDH5MBPbIqV92AaeXatLxBI9gBaebbnrfifHhDYfgasaacH8akY=wiFfYdH8Gipec8Eeeu0xXdbba9frFj0=OqFfea0dXdd9vqai=hGuQ8kuc9pgc9s8qqaq=dirpe0xb9q8qiLsFr0=vr0=vr0dc8meaabaqaciaacaGaaeqabaqabeGadaaakeaadaWcaaqaaiabdcfaqjabcIcaOiabdIfayjabgQIiilabdMfazjabcMcaPaqaaiabdcfaqjabcIcaOiabdIfayjabcMcaPiabgEHiQiabdcfaqjabcIcaOiabdMfazjabcMcaPaaaaaa@3CC4@, that is, the confidence of the rule divided by the support of the consequent. Any rule with an improvement less than one does not indicate a real correlation between antecedent and consequent. On the contrary, when improvement is greater than one the resulting rule is better at predicting the consequent.

### Association rules and transaction databases in gene expression data analysis

What exactly constitutes an item or a transaction depends on the application and on the type of information to be extracted. In the present work ARD was applied to extract associations among gene annotations and expression patterns, integrating in this way biological information with experimental data. To extract the type of associations in which we were interested in, transactions are represented by genes and the set of experiments in which each gene is over- or under-expressed represent the itemset (see Table 1a). In this way, gene characteristics can also be properly included into the itemsets. Using the constraint that gene annotations are used as antecedent, this transaction database can be mined to extract associations on the form of: {*gene annotation X *→[+]*condition *1, [+]*condition *2, [-] *condition*3}, which means that "most of the genes annotated with the characteristic *X *were over-expressed in experimental conditions 1 and 2 and under-expressed in experimental condition 3".

**Table 1:** Transaction databases from gene expression data(a) Transaction database used to extract association rules among gene attributes and expression patterns. (b) Transaction database used to extract association rules among genes.

a	
**Transaction**	**Itemset**

gene A	[+]Exp 1, [+]Exp 2, [-]Exp 3, [+]Exp 4, [+]Exp 5 annotation *X*
gene B	[+]Exp 1, [+]Exp 2, [+]Exp 4, [+]Exp 5, annotation *Z*,, annotation *F*
gene C	[+]Exp 1, [+]Exp 2, [-]Exp 3, [+]Exp 4, [+]Exp 5, [+]Exp 6, annotation *X*
gene D	[+]Exp 4, [-]Exp 6, annotation *B*, annotation *C*
gene E	[+]Exp 1, [+]Exp 2, [-]Exp 3, annotation *X*, annotation *Z*
gene F	[+]Exp 1, [+]Exp 2, [-]Exp 3, [+]Exp 6, annotation *X*, annotation *D*
...	...

b	

**Transaction**	**Itemset**

Experiment 1	[+]gene A, [+]gene B, [+]gene C, [+]gene E, [+]gene F
Experiment 2	[+]gene A, [+]gene B, [+]gene C, [+]gene E, [+]gene F
Experiment 3	[-]gene A, [-]gene C, [-]gene E, [-]gene F
Experiment 4	[+]gene A, [+]gene B, [+]gene C, [+]gene D
Experiment 5	[+]gene A, [+]gene B, [+]gene C
Experiment 6	[+]gene C, [-]gene D, [+]gene F
...	...

Note that this transaction dataset considerably differs from the transaction datasets used in previous applications. As mentioned in the introduction, ARD has been previously used to mine gene expression datasets in order to discover associations among subsets of genes based on their expression information [9-13]. The rules that were proposed in these approaches were on the following form: {[+] *gene A *→[+] *gene B*, [+]*geneC*}, meaning that in a significant number of experiments genes A, B and C are simultaneously over-expressed and whenever Gene A is over-expressed it is likely that Gene B and C are also over-expressed. Table 1b shows the type of transaction database that is generated from a gene expression dataset to extract this type of rules. In this case, transactions are represented by experiments and the subset of over- or under-expressed genes in each experiment represents the set of items (itemset) associated to each transaction.

To construct the transaction database from an annotated dataset the expression matrix has to be previously transformed into a Boolean matrix. For this purpose one can use statistical methods to detect differentially expressed genes or alternatively use a threshold value [10,11]. In this particular application, we have used two expression thresholds in both datasets; genes with log expression values greater than 1 were considered as over-expressed and genes with log expression values lower than the -1 were considered under-expressed. Values between these two ranks were neither expressed nor inhibited. We used these thresholds because they represent an expression or inhibition of two-fold range, which is very often considered significant in microarray studies, and related studies have applied a similar threshold in the analysis of these datasets [36,40]. ARD is a method orientated to discover biological information from gene expression data that has to be applied as a post-analysis procedure. Although users can use arbitrary expression thresholds on fold-changes our recommendation is to determine the expression thresholds by any of the available tools to determine differentially expressed genes before extracting association rules.

### Mining association rules

Given a set of transactions *S*, the problem of mining association rules is to generate all associations that have support, confidence and improvement greater than the user-specified minimum threshold values. The first and key step in the generation of association rules is to find sets of items (itemsets) that satisfy the minimum support constraint (frequent itemsets) on the database. Once the frequent items are located, the subsequent rules can be formed straightforwardly among them.

To effectively generate association rules based on all possible combinations of items, Agrawal proposed the Apriori algorithm [41]. The rationale of this method is to reduce the number of frequent candidate items used for creating the rules by eliminating those that do not satisfy a minimum frequency constraint. The Apriori solution is based on the premise that all subsets of frequent items, as well as their combinations, must also be frequent to be considered as candidates. This property is used to prune the number of candidate itemsets to be explored and thus gaining a significant reduction of the search space. The procedure starts by counting all items with cardinality *k *= 1 and determining the frequent *k*-itemset (formed by individual items with support greater than a given threshold). In the second iteration (*k *= 2) the set of frequent items found in the previous step is used to produce the new set of candidates of size *k*, and the database is scanned again to explore each transaction, and count the frequency of each pair, eliminating those that do not satisfy the minimum support constraint. The procedure is continued until no more combinations are possible.

The type of association rules that we are interested in the context of this work are associations that usually have low support values and, ideally, high confidence values. This is due to the number of genes annotated into a given category typically represents only a small subset of the entire genome which decreases, indeed, when gene annotation specificity increases. The computational problems then arise when trying to use the classical Apriori method to discover associations in datasets that are prolific in frequent patterns at very low-support values. Dropping the support threshold in Apriori algorithm has a severe impact on the amount of CPU time required which it is not affordable in most of the cases because the time needed to solve this problem grows exponentially as the threshold support decreases due to the growth of the combinatorial search space. The apriori algorithm and its many improved variants have concentrated efforts on optimizing the counting of candidate frequency through the use of many clever strategies [42-44] that usually works very well on several applications and that can certainly be used in the one described here. In this work we decided to use a particular algorithm that avoids the expensive combinatorial search space generation by using a novel approach for item counting [45]. This algorithm has already been proved to be very efficient when applied to sequence analysis, motivating us to extend it to gene expression mining, in particular for searching high confidence, low-supported biologically significant patterns. The implementation of the algorithm allows us to specify what type of items should be in the antecedent or the consequent of the rule. Once the rule composition is defined, our method generates all associations that satisfy the minimum support, confidence and improvement constraints.

### Filtering rules

Even if ARD provides quite interesting and important information about local features in the dataset, it has two important drawbacks. First, the number of generated rules, even from small datasets, is already too large to be manually analyzed. Second, many of the extracted rules contain redundant information. Obviously, these two problems are directly related. In this sense, a filter option based on the observed redundancy was implemented to process the obtained rules. The filter conditions proposed to reduce the set of generated rules are the following:

- *Redundant filter*: This option filters out all those rules that are redundant. We consider a rule *X *as a redundant association if there is another rule *Y *with equal or higher values for support, confidence and improvement and:

(i) The consequent and antecedent of *X *is contained in the consequent and antecedent of *Y *respectively.

(ii) The consequent of *X *is contained in the consequent of *Y *and the antecedent part of both rules is the same.

(iii) The antecedent of *X *is contained in the antecedent of *Y *and the consequent part of both rules is the same.

For example, for the rules;

*j*: {*annotation A *→[+]*condition *2},

*x*: {*annotation A *→[+]*condition *2, [+]*condition *6},

*y*: {*annotation B *→[+]*condition *2, [+]*condition *6} and

**Figure 6:**
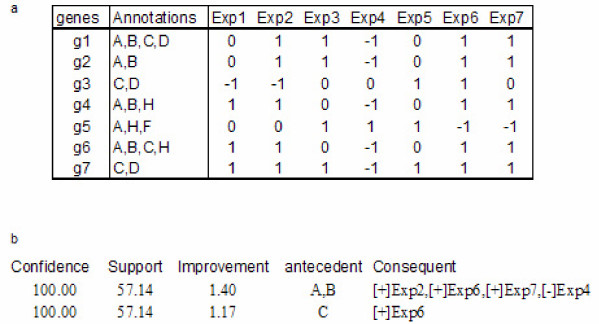
**Example of dataset containing heterogeneous information and the two obtained rules. **(a) Example of dataset in which genes (named as g1, g2...) are annotated with different characteristics (second column). Third and the rest of columns represent experimental conditions and values of 1 represent over-expression, -1 under-expression and 0 neither expression nor inhibition. (b) Two rules that were selected after applying the filter.

*z*: {*annotation A and B *→[+]*condition *2, [+]*condition*6},

we consider the rules *j*, *x *and *y *as redundant associations if their values for support, confidence and improvement are equal or less than the corresponding values of *z*. The rule with the longest consequent or antecedent summarizes all information, and the rest of the rules can be discarded. This pruning method drastically reduces the number of associations to be further considered. This fact is illustrated in Figure 6, in which a small Boolean dataset was mined for association rules using minimum support of 50% and minimum confidence of 80%. These constraints generated 42 associations but after applying this filter option only two rules remained for further analysis.

This notion of redundant associations is different with respect to the notion of insignificant associations [46]. Insignificant association rules are those that contain little extra information while redundant rules do not contain any additional information. Indeed, the insignificant rules pruning method can be applied as a subsequent step to redundant filter option proposed in this work.

- *Single antecedent filter*: This option filters out all rules whose antecedent contains more than one item. This option has been previously proposed by other authors and it can be useful in many contexts, for example when we are looking for information about which subsets of genes (consequent) are over- or under-expressed depending on the expression of a single gene (antecedent) [10].

### Statistical significance of extracted rules

Although support and improvement values provide information about the association between the antecedent and consequent parts of the rule, they do not inform about their statistical significance [39]. The statistical significance of an association was evaluated here using the influence of statistical dependency between the antecedent and the consequent of the rule. For this purpose we used the χ^2^- test for statistical independence [39,46]. A *p*-value associated to each rule was computed under the assumption that the null hypothesis of the test is true (both the antecedent and consequent part of the rule are independent). As we are simultaneously analyzing multiple associations the obtained *p*-values need to be adjusted to avoid the multiple testing problem. To this end we have used a permutation test to correct *p*-values [47,48]. We randomized the gene expression data [49] and association rules were extracted from this random data with no confidence and improvement thresholds. *P*-values were calculated as described above. This process was repeated using 1000 independently generated random datasets. Adjusted *p*-values were calculated for each association in real data as the fraction of permutations having any association with a *p*-value as good or better than the observed *p*-value for that association [47]. Usually a cutoff of 0.05 on the adjusted p-vales can be more safely used to consider an association statistically significant.

### Implementation of ARD

The method was implemented as a web-based tool and is included in the Eng*e*ne™ (Gene Expression Data Processing and Exploratory Data Analysis) software package [17], freely accessible upon request. Gene expression dataset can be uploaded as a standard microarray data format, in which genes are in rows and experiment are in columns. Additional columns can be added to include several gene annotations for each gene. The extraction of association rules involves two steps, the extraction of the transaction dataset followed by the process of discovering association rules. The program allows users to define the rule composition by specifying what type of information should appear in the antecedent and in the consequent of the rule. Detailed information is available on the Eng*e*ne on-line help.

### Gene expression datasets and annotation of gene characteristics

#### Diauxic shift dataset

Gene expression matrix containing background-corrected ratios was downloaded from ExpressDB [50] and were log scaled (base 2). Missing values were filled by k-nearest neighbors approach with *k *= 10 [51] and gene expression profiles of replicated ORFs were averaged.

Yeast genes were annotated using two different gene characteristics; metabolic pathway(s) in which each gene is involved and transcriptional regulators that binds to promoter regions. Metabolic pathways were attached to each gene based on the information provided by the KEGG database [20]. Yeast transcriptional regulators that bind to promoter region were annotated using data reported by Lee *et al*. [21]. This information was used to annotate yeast genes whose promoter regions were bound by at least one transcription regulator (with a *p*-value threshold of 0.005). A relaxed *p*-value threshold can include false positives in regulator-DNA interactions data. For this reason, genome-wide location data have been previously analyzed using relatively stringent *p*-value threshold (0.001) at the expense of losing regulator-DNA interactions [21]. Nonetheless, integration of genome-wide location with expression data allow the use of more relaxed p-value thresholds with less likelihood of false regulator-DNA interactions results [23].

#### Serum stimulation dataset

Iyer *et al*. (1999) characterized the temporal program of gene expression of human fibroblasts after serum exposure. In their experiment they monitored expression levels of 9706 cDNAs, which represented about 8600 different human genes, at 12 times ranging from 15 minutes to 24 hours after serum stimulation. Using this dataset Iyer *et al*. identified 517 genes whose expression changed substantially in response to serum. This gene expression dataset is available at [52]. Gene expression values were log scaled (base 2) and gene expression profiles of replicated genes were averaged.

The Onto-Miner program [53] was used to annotate all genes in the array with the corresponding terms from the three categories of Gene Ontology.

## Authors' contributions

PCS and MC carried out the computational studies and analysis. PCS, AR, APM and OT designed and programmed the association rules discovering algorithm, filters and statistical methods. JMC and APM managed and coordinated the project. All authors participated in writing, approving and revising the final manuscript.

## Supplementary Material

Additional File 1 **Association rules from the diauxic shift dataset **Excel file containing the set of rules obtained before and after filtering from the three analyses of the diauxic shift dataset. Click here for file

Additional File 2 **Association rules from the serum stimulation dataset **Excel file containing the set of rules obtained before and after filtering from the two analyses of the serum stimulation dataset Click here for file
